# Taxonomy, chemodiversity, and chemoconsistency of *Aspergillus*, *Penicillium,* and *Talaromyces* species

**DOI:** 10.3389/fmicb.2014.00773

**Published:** 2015-01-12

**Authors:** Jens C. Frisvad

**Affiliations:** Section of Eukaryotic Biotechnology, Department of Systems Biology, Technical University of DenmarkKongens Lyngby, Denmark

**Keywords:** *Aspergillus*, *Penicillium*, *Talaromyces*, secondary metabolites, chemodiversity, chemoconsistency

## Abstract

*Aspergillus*, *Penicillium,* and *Talaromyces* are among the most chemically inventive of all fungi, producing a wide array of secondary metabolites (exometabolites). The three genera are holophyletic in a cladistic sense and polythetic classes in an anagenetic or functional sense, and contain 344, 354, and 88 species, respectively. New developments in classification, cladification, and nomenclature have meant that the species, series, and sections suggested are natural groups that share many extrolites, including exometabolites, exoproteins, exocarbohydrates, and exolipids in addition to morphological features. The number of exometabolites reported from these species is very large, and genome sequencing projects have shown that a large number of additional exometabolites may be expressed, given the right conditions (“cryptic” gene clusters for exometabolites). The exometabolites are biosynthesized via shikimic acid, tricarboxylic acid cycle members, nucleotides, carbohydrates or as polyketides, non-ribosomal peptides, terpenes, or mixtures of those. The gene clusters coding for these compounds contain genes for the biosynthetic building blocks, the linking of these building blocks, tailoring enzymes, resistance for own products, and exporters. Species within a series or section in *Aspergillus*, *Penicillium,* and *Talaromyces* have many exometabolites in common, seemingly acquired by cladogenesis, but some the gene clusters for autapomorphic exometabolites may have been acquired by horizontal gene transfer. Despite genome sequencing efforts, and the many breakthroughs these will give, it is obvious that epigenetic factors play a large role in evolution and function of chemodiversity, and better methods for characterizing the epigenome are needed. Most of the individual species of the three genera produce a consistent and characteristic profile of exometabolites, but growth medium variations, stimulation by exometabolites from other species, and variations in abiotic intrinsic and extrinsic environmental factors such as pH, temperature, redox potential, and water activity will add significantly to the number of biosynthetic families expressed in anyone species. An example of the shared exometabolites in a natural group such as *Aspergillus* section *Circumdati* series *Circumdati* is that most, but not all species produce penicillic acids, aspyrones, neoaspergillic acids, xanthomegnins, melleins, aspergamides, circumdatins, and ochratoxins, in different combinations.

## INTRODUCTION

The genera *Aspergillus sensu lato* and *Penicillium sensu lato* contain a high number of very diverse species. These species produce a large number of exometabolites, also known as secondary metabolites. Exometabolites are small molecules produced during morphological and chemical differentiation that are outward directed, i.e., secreted or deposited in or on the cell wall, and accumulated in contrast to endometabolites (primary metabolites), that are fluctuating in concentration (the fluxome), and either transformed into other endometabolites or feeding into exometabolites, exoproteins, exopolysaccharides, and morphological structures. While endometabolites can be found in almost all species of fungi (and most other kinds of organisms), exometabolites, exoproteins, and exopolysaccharides are taxonomically restricted, being produced in species-specific profiles. Some metabolites can occur both as endo- and exometabolites, for example citric acid. When citric acid is part of the mitochondrial fluxome, it should be regarded as an endometabolite, but when citric acid is secreted and accumulated (Goldberg et al., 2006; Andersen et al., 2011; Poulsen et al., 2012), as in *Aspergillus niger*, it must be regarded as an exometabolite. Accumulation of citric acid requires that there is a reductive pathway for it in the cytosol and that it can be secreted to the surroundings via an exporter. Thus the transport from the mitochondria to the cytosol, the cytosolic reduction, and the secretion requires a dedicated gene cluster. Such a gene cluster has been found in for example *A. terreus* that is coding for accumulating and secreting itaconic acid (van der Straat et al., 2014), but the gene cluster for citric acid accumulation has not been described yet. Some species related to *Aspergillus* and *Penicillium*, such as *Xeromyces bisporus*, are predominantly stress-selected (S-selected) and the lack of any competitors at very low water activities will have the consequence that *X. bisporus* produces no exometabolites (Leong et al., 2014). In *Aspergillus* most species produce a large number of exometabolites, but some stress selected species, such as *A. penicillioides* and *A. restrictus*, have only been reported to produce asperglaucide and cristatin A, and the related arestrictin A and B (Itabashi et al., 2006). However, the closely related xerotolerant/xerophilic species in the *Aspergillus* subgenus *Aspergillus* (formerly *Eurotium*) produce a high number of exometabolites in the ascomata, making them chemically very diverse (Slack et al., 2009).

## *Aspergillus* AND *Penicillium* TAXONOMY AND NOMENCLATURE

Because of their importance, species of *Aspergillus* and *Penicillium* have been taxonomically treated several times, but the monographs by Raper and Thom (1949) for *Penicillium* and Raper and Fennell (1965) for *Aspergillus* are still regarded as cornerstones in the taxonomy of these fungi. In the period between these two monographs, however, several authors (Benjamin, 1955; Malloch and Cain, 1971) suggested to use names for the sexual state of the Aspergilli and Penicillia, whenever possible, to adhere to the Botanical Code in a nomenclatural sense. The use of *Penicillium* and *Aspergillus* for species that had not yet been found to produce a sexual state could keep their *Penicillium* and *Aspergillus* names, because of a special nomenclatural “exception” in the Botanical Code (Art. 59) that allowed to use two names for a specific fungal species, one for the asexual states (the “anamorph”) and one for the sexual state (the “teleomorph”). Despite this it was recommended to use the sexual name for the whole fungus (the “holomorph”), whenever a sexual state had been found. For this reason many species in *Penicillium* were renamed *Eupenicillium* or *Talaromyces* (Pitt, 1980) and many *Aspergillus* species were renamed *Chaetosartorya*, *Emericella*, *Eurotium, Fennellia, Hemicarpenteles*, *Hemisartorya*, *Neocarpenteles*, *Neopetromyces*, *Neosartorya*, *Petromyces, Saitoa*, *Sclerocleista*, or *Warcupiella* (Rai and Chowdhery, 1975; Rajendran and Muthappa, 1980; Gams and Samson, 1986). To give one example of the name changes one can mention a fungus that was originally described as *A. fischeri*. Since this fungus was described including the sexual state, it could not be used for the asexual state anymore, so the correct name for the fungus according the nomenclatural rules before 2011 was *Neosartorya fischeri*, while *A. fischeri* had to be renamed *A. fischerianus* if one wanted only to refer to the asexual state. A full monographic revision of *Aspergillus* according to the Botanical Code has not been written, but lists of accepted *Aspergillus* and *Penicillium* species have been made (Pitt et al., 2000) and several revisions of the individual genera have been published.

In 2010 it was suggested to introduce a new nomenclatural system in which one fungus had only one name (Hawksworth et al., 2011; Hawksworth, 2012). This suggestion was adopted by the Botanical Congress in Melbourne (McNeill et al., 2012), and thus hereafter any species in fungi will only have one official name. The selection of those names is encouraged to take place by consensus among international experts in the group of fungi under consideration. The International Commission of *Penicillium* and *Aspergillus* (ICPA) has decided to use *Penicillium* for the monophyletic clade that includes *Penicillium* subgenera *Aspergilloides*, *Furcatum,* and *Penicillium sensu* Pitt (1980), *Eupenicillium*, *Chromocleista*, *Thysanophora* and *Eladia,* and *Talaromyces* for the monophyletic clade that includes *Talaromyces* itself and *Penicillium* subgenus *Biverticillium sensu* Pitt (1980). For *Aspergillus sensu lato,* a cladistic study using DNA sequence data, showed that most known *Aspergillus* species were included in a monophyletic clade (Houbraken and Samson, 2011), while a few rare species, such as *A. zonatus* and *A. clavatoflavus* were more closely related to other genera in the *Eurotiomycetes*. The nomenclatural consequence of this is to call all the species in the monophyletic clade *Aspergillus* (Houbraken et al., 2014; Samson et al., 2014) or retypify the genus *Aspergillus* with for example *A. niger*, and then subdivide *Aspergillus* into the genera *Aspergillus*, *Neosartorya*, *Emericella*, *Eurotium,* and *Chaetosartorya* (Pitt and Taylor, 2014). This would have the consequence that the name *Aspergillus* would only be used for a paraphyletic weakly supported clade representing subgenus *Aspergillus* and that the genus *Neosartorya* would be polyphyletic as it includes *Dichotomomyces*. Even though a majority of ICPA members voted for the *Aspergillus* solution, which includes mentioning the sexual state informally, for example *A. fischeri* (neosartorya-morph present), general consensus has not yet been reached. In this review *Aspergillus* names will be used, as suggested by Samson et al. (2014), as the name *Aspergillus* can be confidently used for the monophyletic clade that includes the genera listed above (Houbraken et al., 2014). All species formerly included in *Dichotomomyces*, *Cristaspora, Phialosimplex*, *Polypaecilum*, in addition to *Penicillium inflatum*, have been formally combined into *Aspergillus* (Samson et al., 2014), while *A. crystallinus*, *A. malororatus,* and *A. paradoxus* (*Hemicarpenteles paradoxus*) have been combined into *Penicillium*, as *P. crystallinum*, *P. malodoratum,* and *P. paradoxum* (Visagie et al., 2014b). This means that the presence of aspergilla in an isolate does not necessarily mean that the isolate belongs to *Aspergillus sensu stricto*, and the presence of penicilli in an isolates does not necessarily mean the species belong in *Penicillium sensu stricto*. However, in the majority of cases aspergilla or penicilli indicates that the species belong to *Aspergillus* and *Penicillium*, respectively.

Pitt and Taylor (2014) suggested to use *Aspergillus* for the paraphyletic subgenus *Circumdati* only (after potential re-typification of *Aspergillus* with *A. niger*), stating that this restricted use of the genus *Aspergillus* would make this genus phenotypically different from the closely related *Aspergillus* subgenus *Nidulantes* and therefore suggested the name *Emericella* for the latter monophyletic clade. However, there are many phenotypic traits in common between section *Circumdati* and *Nidulantes*, including the presence of hülle cells and the exometabolites kojic acid, aflatoxins, and sterigmatocystins in both subgenera (Raper and Fennell, 1965; Wiley and Simmons, 1973; Frisvad and Samson, 2004a; Frisvad et al., 2005; Zalar et al., 2008). Subgenus *Circumdati* includes species with both multiple cleistothecia in sclerotia (*Petromyces, Neopetromyces, Saitoa*; Udagawa et al., 1994; Yaguchi et al., 1994; Frisvad and Samson, 2000; Horn et al., 2013) and pseudoparenchymatous multiple ascomata in hyphal masses with or without hülle cells (*Fennellia* and the perfect state of *A. terreus*; Wiley and Simmons, 1973; Locquin-Linard, 1990; Yaguchi et al., 1994; Samson et al., 2011a; Arabatsis and Velegraki, 2013), while several species in *Aspergillus* section *Nidulantes* produce pseudoparenchymatous single ascomata and hülle cells.

Assistance in choosing between *Aspergillus* (Samson et al., 2014) versus the genera *Eurotium, Emericella, Neosarotrya* and *Chaetosartorya, Phialosimplex, Polypaecilum*, *Dichotomomyces,* and *Cristaspora* (Pitt and Taylor, 2014) can be sought from scientific databases. It is very clear that while *Aspergillus* has been used in 56178 publications the other genera, when all added, have only been used in 1093 publications (approximately 2%; **Table 1**).

**Table 1:** References to *Aspergillus, Penicillium, Talaromyces,* and associated genera (Web of Science, as of 18 October, 2014).

Genus	No hits in web of science
*Aspergillus*	56178
*Penicillium*	18011
*Talaromyces*	645
*Eurotium*	421
*Emericella*	379
*Neosartorya*	278
*Eupenicillium*	187
*Thysanophora*	35
*Petromyces*	31
*Dichotomomyces*	12
*Fennellia*	10
*Basipetospora*	9
*Polypaecilum*	7
*Chaetosartorya*	6
*Eladia*	5
*Hemicarpenteles*	5
*Phialosimplex*	5
*Warcupiella*	4
*Neopetromyces*	4
*Chromocleista*	4
*Sclerocleista*	3
*Saitoa*	1
*Neocarpenteles*	1
*Cristaspora*	1
*Hemisartorya*	0

A classification of isolates into sections and series in *Penicillium*, *Talaromyces,* and *Aspergillus* based on phenotypic characters will show that these supraspecific taxa are natural polythetic classes (Beckner, 1959) in exometabolite, ecophysiological, and morphological characters. For example in *Aspergillus* subgenus *Circumdati* section *Circumdati* (the former *A. ochraceus* group) most species, but not all, produce aspyrones, penicillic acids, xanthomegnins, ochratoxins, melleins, circumdatins, neoaspergillic acids, and aspergamides/stephacidins (Frisvad et al., 2004a,b; Finefield et al., 2012; Visagie et al., 2014a). In addition individual species produces exometabolites that are only accumulated by few species in the section. *A. westerdijkiae* and *A. ochraceus* can both produce all the exometabolites listed above, but in addition *A. westerdijkiae* produces preussin and mellamide, not produced by *A. ochraceus*.

At present *Aspergillus* comprises 344 species (Samson et al., 2014), *Penicillium* 354 species (Visagie et al., 2014b), and *Talaromyces* 88 species (Yilmaz et al., 2014). These genera include species that have been reported to produce large numbers of exometabolites (**Table 2**).

**Table 2:** Individual exometabolites produced by important genera of filamentous fungi ranked according to highest number of exometabolites reported (according to AntiBase).

Genus	Number of exometabolites reported
*Aspergillus*	1984
*Penicillium*	1338
*Fusarium*	507
*Trichoderma*	438
*Talaromyces*	316
*Phoma*	263
*Drechslera, Curvularia, Bipolaris, Cochliobolus*	258
*Alternaria* and *Ulocladium*	231 + 7
*Chaetomium*	230
*Acremonium*	187
*Phomopsis*	186
*Xylaria*	143
*Stachybotrys*	138
*Pestalotiopsis*	133
*Claviceps*	130
*Cladosporium*	113
*Botrytis*	102
*Byssochlamys/Paecilomyces sensu stricto*	94
*Hypoxylon*	88
*Cordyceps*	77
*Clonostachys*	72
*Arthrinium*	26
*Nigrospora*	25
*Septoria and Stagonospora* and *Parastagonospora*	22
*Stemphylium*	17
*Trichophyton*	10

*Species of *Penicillium* listed were revised to *Talaromyces* if they belonged there (Yilmaz et al., 2014).*

## CHEMODIVERSITY

Species of *Aspergillus*, *Penicillium,* and *Talaromyces* are extraordinarily productive concerning exometabolites. A comparison with other genera shows that most exometabolites have been reported from *Aspergillus* (1984), next-most from Penicillium (1338), and fifth-most by *Talaromyces*, (316), with only *Fusarium* (507) and *Trichoderma* (438) producing more exometabolites *in toto* (**Table 1**). The number of exometabolites pr species is 5.77 for *Aspergillus*, 3.77 for *Penicillium,* and 3.58 for *Talaromyces*. These number per species are clearly underestimates as some exometabolites are produced by more than one species in a genus, in addition to the fact that many species have not been examined and that some exometabolites are only expressed under unique circumstances and thus may remain undetected (Sanchez et al., 2012; Brakhage, 2013; Scherlach et al., 2013; Takahashi et al., 2013; Bertrand et al., 2014; Marmann et al., 2014). Light, pH, redox potential, temperature, water activity, carbon sources, nitrogen sources, iron starvation, and exometabolites from other species can all have a regulatory effect on the regulatory proteins for exometabolite expression in a fungus (Brakhage, 2013). A majority of the exometabolites produced by *Penicillium* and *Aspergillus* are only found sporadically in other genera, but a large number of exometabolites are in common between *Aspergillus* and *Penicillium*. On the other hand exometabolites from *Talaromyces* are nearly all unique to that genus (Samson et al., 2011b), or only shared with few other species.

The same exometabolite may be produced by widely different species. For example aflatoxin is produced by the species listed in *Aspergillus* section *Flavi* (15 spp.), *Aspergillus* section *Nidulantes* (3 spp.), *Aspergillus* section *Ochraceorosei* (2 spp.), and *Aschersonia* (2 spp.; Frisvad and Samson, 2004a; Frisvad et al., 2005; Zalar et al., 2008; Varga et al., 2009, 2011; Kornsakulkarn et al., 2012, 2013; Massi et al., 2014). Three species in *Aspergillus* section *Flavi* and all the seven species outside section *Flavi* listed above only produce aflatoxins of the B type. It is surprising that aflatoxins have never been found in *Penicillium*, but they have been found in the unrelated scale insect fungi *Aschersonia coffea* and *Aschersonia marginata* (Kornsakulkarn et al., 2012, 2013). However, the precursor sterigmatocystin, although end-product for some species, has been found in a large number of unrelated genera (Rank et al., 2011), suggesting that this complicated gene cluster has been horizontally transfered between species in widely different genera, as shown by Slot and Rokas (2011) for *Podospora anserina* and *A. nidulans*. Fungal species are specifically associated to certain habitats or few plant, animal, or other kind of organisms (Filtenborg et al., 1996), and will therefore produce exometabolites in reponse to the challenges in the particular habitat. For example *P. herquei* was thought to be a soil fungus saprophyte (Kwaśna, 2004), but recent studies have shown tha the leaf-rolling weevil (*Euops chinensis*) have developed mycangia to inoculates leaves with *P. herquei* conidia to protect the weevil eggs (Li et al., 2012). *P. herquei* produce a species specific profile of exometabolites, of which several are antibiotically active (Petit et al., 2009; Tansakul et al., 2014). Thus the specificity in both association of fungal species to other species and the profile of exometabolites are factors that have boosted the evolution of so many exometabolites.

*Dichotomomyces cejpii* was transferred to *A. cejpii* by Samson et al. (2014), and this new combination is supported by chemotaxonomic evidence. *D. cejpii* was reported to produce gliotoxin, xanthocillin X monomethylether, tryptoquivalones, JBIR-03, emindole SB, emindole SB beta-mannoside, and 27-*O*-methylasporyzin (Varga et al., 2007; Harms et al., 2014). While gliotoxin, tryptoquivalones, and xanthocillins (Frisvad et al., 2009; Zuck et al., 2011) indicates a relationship to *A. fumigatus* and tryptoquivalones a close relationship to *A. clavatus*, as supported by DNA sequences (Varga et al., 2007), production of emindole SB indicates a relationship to *Aspergillus* section *Nidulantes*. The report of emindole SB, emeniveol, asporyzin A-C, and JBIR-03 from a marine-derived *A. oryzae* (Qiao et al., 2010), indicates what they identified as “*A. oryzae*” is a fungus related to *A. cejpii* or a species in *Nidulantes* rather than *A. oryzae*, however.

## CHEMOCONSISTENCY AND OSMAC

The abbreviation OSMAC (one strain many compounds) was introduced by Bode et al. (2002) where the authors showed, among several examples, that a strain of *A. westerdijkiae* produced a series of exometabolites that could be ordered into different biosynthetic families. Furthermore, by using several media a more full profile of these exometabolites could be revealed. The idea that one strain can produce several exometabolites was already introduced by Frisvad (1981) and Frisvad and Filtenborg (1983, 1989). These authors showed that terverticillate penicillia produced a unique profile of different exometabolites and also that certain media, such as Czapek yeast autolysate (CYA) agar and yeast extract sucrose (YES) agar were very efficient for production of a large number of different exometabolites, while further media may increase the number of exometabolites expressed (Bills et al., 2008; Nielsen et al., 2011; Frisvad, 2012). Furthermore they showed that these profiles of exometabolites were species specific and consistent from isolate to isolate, i.e., the isolates in anyone fungal species were chemo consistent (Larsen et al., 2005). One of the original terms for exometabolites or secondary metabolites was idiolites, the latter indicating that production of exometabolites was strain specific, however, exometabolite profiles are clearly species specific (Frisvad et al., 2008). However, a single mutation in a gene in an exometabolite gene-cluster will often be sufficient for loss of phenotypic expression (Susca et al., 2014), and this may be the reason some authors call the production of certain exometabolites “strain-specific” (i.e., Engel et al., 1982). The ability to produce mycophenolic acid in *P. roqueforti* is retained in most strains, however, (Frisvad and Filtenborg, 1989; Geisen et al., 2001; Frisvad and Samson, 2004b), but is not as consistent as in *P. brevicompactum*, where a non-producing strain has never been found (Frisvad and Filtenborg, 1989; Frisvad and Samson, 2004b). Reasons for observing “unusual “ or “unexpected” exometabolites in a species may be horizontal gene transfer of a gene cluster in only one or few strains, hybridization (which is not common in filamentous fungi), or epigenetic priming. Raper and Thom (1949) mentioned a strain of *P. citrinum* (NRRL 822, their group III in a subdivision of *P. citrinum* “transitional toward *P. chrysogenum* series”) produced both citrinin, known from this species, and penicillin, known from *P. chrysogenum*. They also mentioned that their strain had the cultural appearance of both *P. citrinum* and *P. chrysogenum*. We have re-examined this strain, and indeed it had characters of both species, and appeared to be a (rare) hybrid. However, exometabolites from co-occurring species from the same habitat may stimulate the epigenome by acting as inhibitors of histone acetylation or methylation, and this exometabolite stimulation will be one of many ways of having silent exometabolite gene clusters in filamentous fungi expressed (Bertrand et al., 2014). It was recently shown that *A. niger* could produce sclerotia with many hitherto not expressed aflavinins in them (Frisvad et al., 2014) simply by stimulating *A. niger* with whole fruits or rice. Whether this stimulation is caused by extrolites from those whole fruits or rice or from a physical stimulation is not yet known. Furthermore variations in the growth medium and ecophysiological factors such as pH, temperature, and water activity will obviously also stimulate expression of gene clusters of exometabolites that were initially thought to be silent.

In conclusion *Aspergillus, Penicillium,* and *Talaromyces* contain species that produce a very large number of species-specific exometabolites with a high degree of chemoconsistency. The chemodiversity of the many species in these three genera is extremely high and many more bioactive compounds from the species will be found in the future. Both ecological and genetic/molecular approaches are needed to fully explore this treasure-trove of natural products.

## Conflict of Interest Statement

The author declares that the research was conducted in the absence of any commercial or financial relationships that could be construed as a potential conflict of interest.
